# Beneficial Effect of Olive Oil and Its Derivates: Focus on Hematological Neoplasm

**DOI:** 10.3390/life14050583

**Published:** 2024-04-30

**Authors:** Chiara Campo, Sebastiano Gangemi, Giovanni Pioggia, Alessandro Allegra

**Affiliations:** 1 Division of Hematology, Department of Human Pathology in Adulthood and Childhood “Gaetano Barresi”, University of Messina, 9815 Messina, Italy; chiaracampo210@gmail.com; 2 School and Operative Unit of Allergy and Clinical Immunology, Department and Experimental Medicine, University of Messina, 98122 Messina, Italy; gangemis@unime.it; 3 Institute for Biomedical Research and Innovation (IRIB), National Research Council of Italy (CNR), 98158 Messina, Italy; giovanni.pioggia@irib.cnr.it

**Keywords:** antioxidant activity, apoptosis, leukemia, multiple myeloma, olive leaf extract, phenolic compounds

## Abstract

Olive oil (*Olea europaea*) is one of the major components of the Mediterranean diet and is composed of a greater percentage of monounsaturated fatty acids, such as oleic acid; polyunsaturated fatty acids, such as linoleic acid; and minor compounds, such as phenolic compounds, and particularly hydroxytyrosol. The latter, in fact, are of greater interest since they have found widespread use in popular medicine. In recent years, it has been documented that phenolic acids and in particular hydroxytyrosol have anti-inflammatory, antioxidant, and antiproliferative action and therefore interest in their possible use in clinical practice and in particular in neoplasms, both solid and hematological, has arisen. This work aims to summarize and analyze the studies present in the literature, both in vitro and in vivo, on the possible use of minor components of olive oil in some hematological neoplasms. In recent years, in fact, interest in nutraceutical science has expanded as a possible adjuvant in the treatment of neoplastic pathologies. Although it is worth underlining that, regarding the object of our study, there are still few preclinical and clinical studies, it is, however, possible to document a role of possible interest in clinical practice.

## 1. Introduction

Numerous epidemiological studies spanning diverse populations and regions have consistently underscored the potential of olive oil as a dietary element in mitigating the risk of cancer [1]. Recognized as a cornerstone of the Mediterranean diet, olive oil (*Olea europaea,* Oleaceae) has garnered attention for its multifaceted health benefits [2]. Taxonomically, *O. europaea* ssp. europaea belongs to the Oleaceae family, which encompasses both cultivated (var. europaea) and wild (var. sylvestris) variants, reflecting its broad genetic diversity and ecological adaptability [1].

Olive oil, available in various forms, is integral to culinary and dietary practices globally. Virgin olive oils, celebrated for their minimal processing that preserves the oil’s natural composition, include extra virgin, virgin, and ordinary virgin olive oils. Conversely, refined olive oils undergo specific transformations before consumption, encompassing lampante virgin olive oil, refined olive oil, blends of refined and virgin olive oils, and pomace oil [3].

Despite these variations, virgin and refined oils exhibit similar fatty acid profiles, with oleic acid constituting the predominant component, complemented by palmitic and linoleic acids, all of which contribute to its nutritional value [4].

The production of olive oil involves a range of extraction methods that directly influence the quality and characteristics of the resulting oil. The extraction process typically begins with the harvesting of olives during their optimal ripening phase. The olives are then cleaned to remove any impurities and plant residues. Subsequently, the olives are crushed to break the cell walls and release the oily content within the tissues. This step can be carried out through traditional methods, such as stone milling, or through more modern techniques, such as mechanical crushing [5].

After crushing, the paste obtained undergoes kneading, a process that promotes the aggregation of oil droplets and facilitates separation from the solid pulp. This phase is crucial for the effective extraction of the oils contained in the olives. Kneading can be carried out at controlled temperatures to preserve the organoleptic characteristics of the oil [5]. 

Next, the paste is subjected to a separation phase through centrifugation. During this process, the various components of the paste, including the oil droplets, are separated based on their density. This allows for the extraction of olive oil, which is the lighter phase, separating it from the denser fractions [5]. 

Finally, the extracted oil may undergo further filtration processes to remove any residual impurities and improve its clarity and stability. These extraction methods may vary slightly depending on the production region and the preferences of the producer, but they remain essential to ensuring the quality and integrity of the final olive oil [5].

Olive oil, a fundamental component of the Mediterranean diet, is characterized by an extremely varied chemical composition. Triglycerides make up the majority of the oil, accounting for about 98–98.5% of its composition. These triglycerides are esters of glycerol and fatty acids, where three molecules of fatty acids are bound to one molecule of glycerol through ester linkages [5]. 

In addition to triglycerides, olive oil contains a wide range of minor compounds, including terpenoids, aliphatic alcohols, sterols, squalene, pigments, tocopherols (vitamin E), and phenolic compounds. 

Terpenoids are organic compounds derived from isoprene units, while aliphatic alcohols are characterized by the presence of a hydroxyl group (-OH) attached to an aliphatic carbon. Sterols, including β-sitosterol and campesterol, are steroid compounds with a hydroxyl group (-OH) at C-3 position and an alkyl side chain. Squalene is an isoprenoid compound that acts as a natural antioxidant in olive oil. Pigments, such as chlorophylls and carotenoids, give the oil its characteristic golden-green color. Tocopherols, also known as vitamin E, are polyphenolic compounds with a hydrocarbon side chain and a phenolic aromatic ring. These tocopherols are lipophilic antioxidants that protect fatty acids from oxidation. Finally, phenolic compounds include molecules with an aromatic ring containing one or more hydroxyl groups (-OH), such as oleuropein and hydroxytyrosol, which provide olive oil with antioxidant and anti-inflammatory properties [5].

Together, these compounds give olive oil a unique chemical complexity that contributes to its sensory, nutritional, and functional properties. It is essential to note that this intricate composition is influenced by various environmental and agronomic factors, such as soil type, climate, and harvesting techniques, which modulate the qualitative and quantitative distribution of its constituents [6]. This variation can have a significant impact on the sensory profile and nutritional properties of olive oil, giving it a unique diversity of aromas and flavors [6].

**Figure 1:**
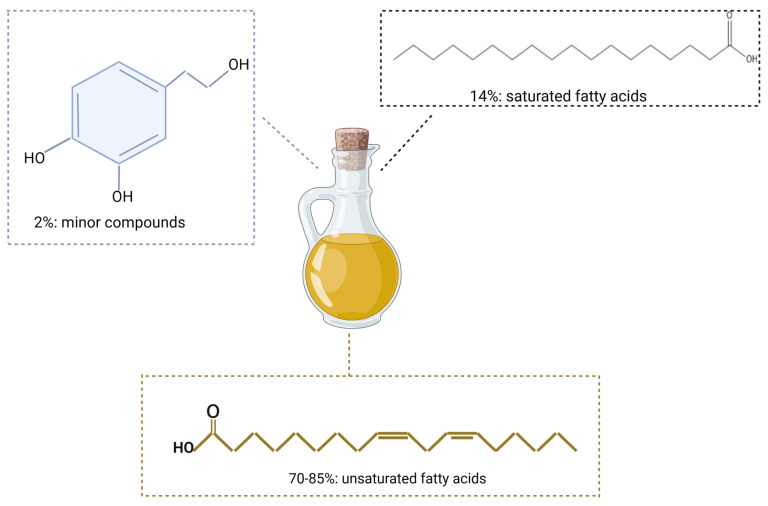
The composition of olive oil: unsaturated fatty acids, saturated fatty acids, and minor compounds. “Created with BioRender.com (accessed on 1 February 2024)”.

Olive oil stands out among vegetable oils for its high content of monounsaturated (MUFA) and polyunsaturated fatty acids, which contribute to its renowned health-promoting properties [7,8]. Oleic acid (C18:1) dominates the unsaturated fatty acid profile, comprising 70–85%, alongside linolenic, arachidonic, gadoleic, behenic, lignoceric, palmitoleic, and heptadecanoic acids, while saturated fatty acids constitute approximately 14% of the oil’s composition [5]. Of particular interest are the minor compounds that constitute approximately 2% of olive oil’s weight, comprising a diverse array of phenolic and lipophilic compounds. Phenolic compounds like hydroxytyrosol and the secoiridoid oleuropein, along with lipophilic compounds such as α-tocopherol (vitamin E) and carotenoids, confer additional health benefits and contribute to the oil’s stability and flavor profile [9,10,11] (Figure 1).

Beyond its culinary and nutritional significance, *Olea europaea* and its extracts have a rich history of medicinal use in traditional and contemporary healthcare systems. Documented applications include hypotensive, emollient, and therapeutic roles in managing lower urinary tract infections [12]. Recent research has advanced hypotheses suggesting that the cancer-preventive effects of olive oil may be attributed, at least in part, to its minor constituents, which encompass over 230 chemical compounds [13]. The beneficial properties of extra virgin olive oil, in particular, have been ascribed to its rich phenolic content, including compounds such as tyrosol, hydroxytyrosol, and their secoiridoid derivatives like oleuropein aglycone and oleocanthal [14,15]. These secoiridoid derivatives are formed during olive oil production processes, specifically crushing and malaxing, facilitated by the activation of the β-glucosidase enzyme. However, their inherent instability in olive oil underscores the importance of production processes and storage conditions in maintaining their potency [16].

The concentration of these minor compounds in olive oil exhibits considerable variability (ranging from 50 to 800 mg/kg) and is influenced by a multitude of factors, including agronomic practices and technological aspects of olive oil production. Consequently, the European Union has implemented regulations to categorize olive oil into distinct types based on production methods and processing techniques, ensuring industry standardization and consumer protection [17]. This regulatory framework, delineated in Article 6 of Commission Delegated Regulation (EU) No 1308/2013, provides a comprehensive classification system aimed at promoting transparency and quality within the olive oil sector [17].

## 2. Materials and Methods

We conducted a comprehensive search of the relevant literature using electronic databases such as PubMed and Google Scholar. The search terms included the following keywords “antioxidant activity”, “apoptosis”, “leukemia”, “multiple myeloma”, “olive leaf extract”, and “phenolic compounds”. Studies were included based on predefined criteria. Inclusion criteria comprised research articles, observational studies, clinical trials, and meta-analyses investigating the association between olive oil intake and hematological neoplasms. Exclusion criteria included studies with irrelevant outcomes, animal studies, and studies not available in English.

## 3. Bioactivity of Olive Oil’s Secoiridoid Derivatives

Numerous studies have demonstrated the crucial role that phenolic compounds play, such as antioxidants, anti-inflammatory agents, and in the control of epigenetic alterations that lead to antiproliferative protection, through miRNA expression, DNA methylation, and histone modifications [7,18,19]. It is widely recognized that in a multistage carcinogenesis model, oxidative stress may not only be related to onset but also to promotion and progression. Reactive oxygen species are known to activate a number of signal transduction pathways, including nuclear factor-kB and activator protein-1, which then trigger the transcription of genes involved in controlling cell development pathways [20]. The antioxidant properties of these substances include their ability to inhibit lipid peroxidation, to act as free radical scavengers, to compete in the electron transport chain with coenzyme Q, and to promote ROS activity. Detoxifying enzymes include superoxide dismutase (SOD), catalase (CAT), glutathione reductase (GSR), and glutathione S-transferase [21,22,23]. The degree of hydroxylation and the presence of a sugar moiety both affect how effective polyphenols are as antioxidant chemicals. Additionally, it greatly depends on their hydroxyl groups’ redox characteristics, the possibility of electron delocalization throughout the chemical structure, and their hydroxyl groups. For instance, the antioxidant capacity of ortho- and para-diphenolics rises when hydrogen atoms are replaced with ethyl or n-butyl groups [22,24].

A recent meta-analysis of randomized controlled trials has also demonstrated that frequent consumption of olive oil reduces inflammation [21,22,23]. In particular, these studies have shown that three plasmatic inflammatory mediators (tumor necrosis factor, IL-6, and C-reactive protein) were decreased [25,26,27]. In different in vitro experiments, using human monocytic THP-1 cells, hydroxytyrosol inhibited the production of pro-inflammatory cytokines (COX-2), reduced the expression of cyclooxygenase-2 (COX-2), induced nitric oxide synthase (iNOS), and inhibited pro-inflammatory cytokines (NF-kB) [25,28]. In an in vitro platelet-rich plasma model, hydroxytyrosol reduced the synthesis of thromboxane B2 (TXB2) [22,25]. In this regard, these authors discovered a decrease in the production of thromboxane B2, which serves as a marker for the synthesis of thromboxane A2 (TXA2). The primary cause of this decline was the inhibition of the cyclooxygenase enzyme’s activity [25,29,30]. Additionally, hydroxytyrosol indirectly inhibited the iNOS and COX-2 enzymes and the following production of prostaglandin E2. This result is due to the inhibition of NF-kB transcriptional activation, interferon regulatory factor-1 activation, and transducer and activator of transcription 1a activation, which inhibited the activation of J774 murine macrophages [25,31]. In fact, the effect of hydroxytyrosol that caused the inhibition of COX-2 and 5-lipoxygenase transcription and the reduction of prostaglandin E2 synthesis could be related to the decrease in chronic diseases like cancer [25,32].

**Figure 2:**
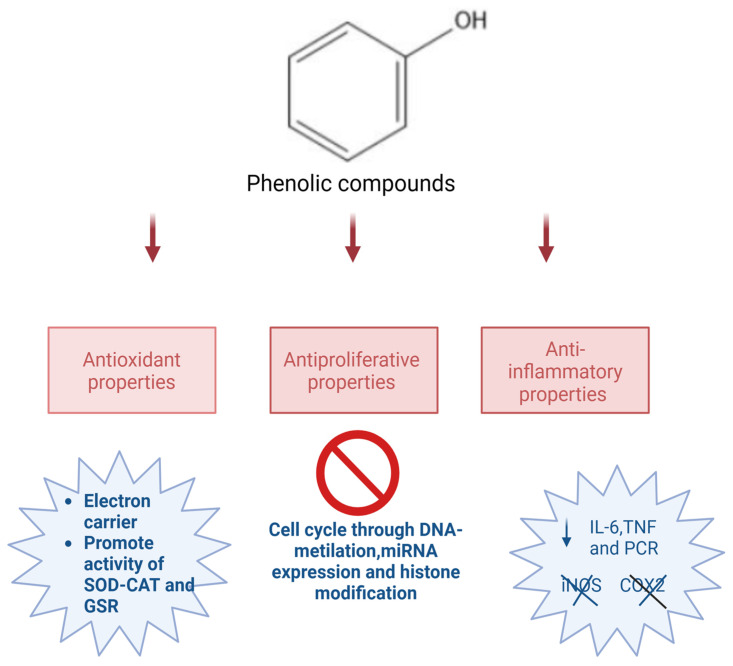
Properties of phenolic compounds and their role demonstrated in different studies. “Created with BioRender.com (accessed on 1 February 2024)”.

The most prevalent MUFA in olive oil, oleic acid, may also help to create a molecular microenvironment that would prevent the onset and growth of tumors [33]. Peroxisome proliferator-activated receptors (PPARs), which can be activated by unsaturated fatty acids like oleic acid, have been demonstrated to have a range of regulatory abilities, including the ability to reduce nuclear factor kappa beta (NF-kB)-related expression, which lowers low-grade inflammation [33,34]. Additionally, oleic acid can alter the expression of several human cancer-related genes, most notably by causing the transcriptional suppression of the HER2 gene [29,32,33,35]. Antioxidant phytochemicals included in virgin olive oil may prevent the growth of cancer by directly scavenging reactive oxygen and nitrogen species (RONS) [33]. Mutations are brought about by oxidized bases because nucleic acid polymerases can no longer read them correctly. This process can be caused by RONS and can promote cell proliferation or suppress cell cycle arrest, increasing the chance of developing cancer [36]. Thus, the RONS neutralization by antioxidants may reduce the frequency of DNA mutations [33]. Autophagy abnormalities and mitochondrial dysfunction may also be brought on by RONS. To get rid of damaged mitochondria, a process known as autophagy targets cytoplasmic components and leads to lysosomal destruction and recycling [33]. Numerous human chronic diseases, including cancer, have been linked to autophagy malfunction [37]. In this regard, a significant number of naturally occurring bioactive compounds have the ability to control autophagy by altering signaling pathways and transcriptional factors [38]. Through autophagy or biogenesis, MUFA and n-3 PUFA are able to support mitochondrial turnover [39]. Oleocanthal, on the other hand, has been demonstrated to decrease cellular viability by excessively stimulating the onset of autophagy [40]. In fact, in different in vivo studies, oleocanthal determines apoptosis through different signaling pathways such as activation of both poly-adenosine diphosphate-ribose polymerase and caspase-3, phosphorylation of p53, suppressing activation of p90 ribosomal S6 kinase and extracellular signal-regulated kinases 1/2, and arrest of cell cycle at G1/S phase and cell proliferation [40] (Figure 2). Otherwise, it has been demonstrated that hydroxytyrosol is an anti-angiogenic substance that can block several important angiogenesis-related processes. Hydroxytyrosol has been demonstrated to have inhibitory effects on several molecular targets, such as MMP-9, matrix metalloproteinase 2 (MMP-2), vascular endothelial growth factor receptor-2 (VEGFR-2) phosphorylation, and cyclooxygenase 2 [41]. These actions account for hydroxytyrosol’s anti-angiogenic potential [42].

## 4. Use of Olive Oil and Secoiridoid Derivatives in Studies Performed in Cell Culture

Evidence from epidemiologic research clearly links olive oil consumption to a reduced incidence of various types of cancer, including prostate, breast, larynx, lung, ovary, and colon cancers [43,44,45,46,47]. Although the aforementioned research provides clear indications that virgin olive oil possesses cancer-protective properties, it remains unclear what exactly causes this effect and how it operates [48]. Recently, more attention has been focused on minor components such as phenolic compounds, which exhibit strong antioxidant activity [49,50], as well as several other biological properties [51]. In this study, we investigate the findings of major in vitro and in vivo studies on olive oil compounds and hematological malignancies (Table 1).

## 5. Olive Oil Compounds and Promyelocytic Leukemia

Numerous epidemiologic studies on cell culture have shown that the phenolic compound hydroxytyrosol (HT), found in olive oil, can thwart the carcinogenic process. These compounds may cause cell cycle arrest, which will slow the growth and division of malignant cells and eventually trigger apoptosis [59].

The initial investigation, carried out by Della Ragione et al. [52,60], showed that HT is capable of halting the cell cycle, lowering growth and proliferation, and triggering apoptosis in HL60 (human promyelocytic leukemia cell line) cells at doses ranging from 50 to 100× M. Since tyrosol (TY), 2-(4-hydroxyphenyl)ethanol, did not cause cell growth arrest or death, these actions need the presence of two ortho-hydroxyl groups on the phenyl ring. The release of cytochrome c, which triggers the effector caspase 3, is necessary for the apoptosis that HT causes. These authors also noted that as the release of cytochrome c precedes caspase 8 activation without affecting death receptor activation, including the FAS receptor, the results exclude the death receptor pathway FAS (TNF receptor super family, member 6) [52,60].

However, another study by Fabiani et al. [48] demonstrated that 3,4-DHPEA ([3,4-dihydroxyphenylethanol, Hydroxytyrosol) may also be connected to its capacity to reduce growth, trigger apoptosis, and promote differentiation in various cancer cell lines [61]. Inhibiting the G1/S transition, halting the cell cycle in the G0/G1 phase, and inducing significant apoptotic cell death are all ways that 3,4-DHPEA, at a dosage of 100 i.m., causes the complete arrest of cell growth in human promyelocytic leukemia cells (HL60) [59,61]. Additionally, it was shown that 3,4-DHPEA modifies the expression of crucial proteins that control the cell cycle and apoptosis by down-regulating the expression of cyclin-dependent kinase 6 (CDK6) and up-regulating the expression of CDK inhibitors such as p21WAF1/Cip1 and p27Kip1 [62]. The first phase of the study by Fabiani et al. consisted of the extraction and separation of phenolic compounds from virgin olive oil: olives (Moraiolo cv) were crushed using a hammer crusher; the malaxation process was completed for 40 min at 25 °C; and the oil was extracted by centrifugation using a decanter with water addition [48]. According to Montedoro et al. [63], a phenolic methanolic extract was obtained from virgin olive oil, containing a concentration of 650 mg/kg of total phenols. The extraction process involved separating the phenolic compounds using semi-preparative HPLC [48,64]. Human promyelocytic leukemia cells (HL60), provided by the American Type Culture Collection, were subsequently treated with various doses of phenolic compounds at a temperature of 37 °C and in an atmosphere containing 5% CO_2_. To evaluate apoptosis, propidium iodide (PI) staining combined with flow cytometry analysis was employed, while agarose gel electrophoresis was utilized to analyze DNA degradation induced by various concentrations of hydroxytyrosol in HL60 cells [48,52,60,65,66].

The study outcomes revealed significant perturbations in cell cycle progression, with a blockade of the G1 phase and a consequent increase in the proportion of cells in the G0/G1 phase, accompanied by a decrease in the number of cells progressing through the S and G2/M phases. These findings assume particular significance in light of the antioxidant properties commonly associated with olive oil, suggesting a potential role in tumor prevention. It is known that altered cells often exhibit abnormalities in the regulation of the cell cycle, underscoring the importance of understanding such mechanisms in tumor pathology [59].

Cell cycle progression, which in turn controls cellular proliferation, is governed by the successive activation of specific cyclin-dependent kinases (Cdks) and the selective induction of various Cdk inhibitors [67]. As previously demonstrated with several other phenolic compounds derived from tea [68], the extracted phenolic compound (DPE) might, therefore, exert its antiproliferative effects by directly interfering with these processes, either by inhibiting Cdks or activating Cdk inhibitors. Additionally, DPE might impede other signaling mechanisms involved in cell proliferation. It is noteworthy that DPE exhibits strong antioxidant activity [69], and that some reactive oxygen species (ROS) have been shown to act as crucial intracellular messengers for the mitotic signal transduction of numerous cytokines and growth factors, completely halting HL60 growth and concurrently triggering apoptosis. DPE at a concentration of 100 mmol/L demonstrated the greatest effects, as evidenced by nuclear morphological alterations, the appearance of a subdiploid peak in DNA content through flow cytometry analysis, and DNA fragmentation [70]. Furthermore, a study conducted by Fabiani et al. demonstrated that the proapoptotic effect exerted by hydroxytyrosol was mediated by the ability of 3,4-DHPEA to generate extracellular H_2_O_2_ in the culture medium [61].

Another study conducted by Abaza et al. [71] utilized ethanol extracts of leaves from seven principal Tunisian olive cultivars, namely Chemlali, Chemchali, Gerboui, Sayali, Chétoui, Zarrazi, and Zalmatì. This study demonstrated that the use of ethanol leaf extracts of olive oil can inhibit the proliferation of HL-60 cells [71].

## 6. Olive Oil Compounds and Chronic Myelogenous Leukemia

Olive oil extracts have been demonstrated to have an impact on human chronic myelogenous leukemia K562 cells in a study conducted by Samet et al. [53]. The study utilized air-dried, mixer-ground Chemlali variety olive leaves sourced from the Sfax region of Tunisia [53]. The K562 cell line for human chronic leukemia was obtained from the Riken Cell Bank in Tsukuba, Ibaraki, Japan [53]. Following a 24 h incubation period, olive leaf extract, diluted, was introduced at final concentrations [53]. In accordance with the published research, the study by Samet et al. has demonstrated that cells treated with COLE (Chemlali olive leaf extract) were arrested at G0/G1 on the first and second days of treatment. Long-term COLE incubation (third and fourth day of incubation) revealed a cell cycle arrest at the G2/M phase [53].

Microarray analysis on the third day of treatment indicated a significantly elevated level of CHEK2 gene expression in COLE-treated cells. CHEK2, through the suppression of CDC25B, CDC25A, and CDC25C activity, controls the cell cycle checkpoint arrest [72]. At various times during the cell cycle, the CDC25 proteins activate the Cdk-cyclin complexes, which then cause the cell to enter mitosis. While CDC25C and CDC25B act in G2/M, CDC25A regulates the G1/S transition early in the cell cycle [73]. It is interesting to note that Samet et al.’s findings revealed a decrease in the expression of the CDC25C gene in COLE-treated cells together with an increase in the expression of the CDC25A gene. This finding may help to explain the cell cycle arrest at the G2/M phase seen on the third day of COLE treatment. The differentiation of K562 cells toward the mono-/macrophage lineage was indicated by the treatment with COLE, which elevated the expression of CD14 on the cell surface of treated cells [53]. This postulation finds support in the observed increase in the proportion of CD11b-positive cells, indicative of heightened commitment of K562 cells towards the monocyte/macrophage lineage at the expense of granulocytic differentiation subsequent to COLE treatment. This phenomenon is concomitant with the elevated expression levels of CD14 and CD11b, both characteristic markers of monocyte/macrophage lineage [53].

Additionally, the transient surge in CD41 expression on the initial day of COLE treatment, followed by its subsequent decline, is consistent with the transcriptional activity of the IIb promoter. This promoter, which initiates CD41 transcription, exhibits activity across various stages of myeloid differentiation, including pluripotent myeloid progenitors, early erythropoiesis, megakaryocytic differentiation, and to a lesser extent, monocyte differentiation [74]. Microarray analysis further reveals the up-regulation of IF16 and EGR-1 genes subsequent to COLE treatment. IF16, implicated in the differentiation of human myeloid cells in response to interferon-gamma, and EGR-1, associated with myeloid progenitor development, collectively underscore the impact of COLE treatment on the regulatory mechanisms governing myeloid differentiation [53].

Moreover, COLE administration elicited an increase in the expression of several phagocytosis-related genes, including those associated with AP1G1 and Rab proteins, alongside chemokine genes such as CXCL2, CXCL8 (IL8), and CXCL3 [53]. Conversely, the expression of the apoptosis suppressor BCL2 and caspase inhibitor genes was downregulated, while several proapoptotic genes, including CASP8, CASP6, BID, and DFFA, were up-regulated. Notably, although leukemia cells exhibited morphological changes indicative of apoptosis upon exposure to olive leaves, precise underlying mechanisms remain incompletely understood [75,76]. The observed increase in apoptotic cells starting on day 4 of COLE therapy may be attributed to the programmed death of fully differentiated cells. Importantly, this rise in CD14 expression coincided with a significant decline in cell viability [53].

In COLE-treated cells, genes encoding mitogen-activated protein kinase kinase kinases (MAPKKKs), including MAP3K5, MAP3K2, and MAP3K7, exhibited substantial up-regulation, while MAPK14/p38- and MAP2K5 were down-regulated among differentially expressed genes [77].The MAP kinase cascade, orchestrated by MAPKKKs, governs critical biological processes such as gene expression, cell proliferation, differentiation, survival, and death [77]. Notably, MAP3K5 activates both JNK and p38 mitogen-activated protein kinases, while MAP3K2 preferentially activates JNK [77,78]. COLE treatment appears to predominantly mediate its effects through JNK MAPKs rather than p38 MAPKs, as evidenced by the significant decline in MAPK14/p38- expression [53]. JNK, activated by MAPKKKs, plays a pivotal role in apoptosis pathways by modulating the expression of pro-apoptotic or anti-apoptotic genes [53]. Additionally, MAP3K2 plays a crucial role in the NF-kappaB signaling pathway [79]. COLE treatment upregulates genes involved in positive regulation of NF-kappaB transcription factor activity, I-kappaB kinase NF-kappaB cascade, and I-kappaB phosphorylation [80]. Genes such as TRAF5, TRAF6, and SNIP1, along with other NF-kappaB pathway signal transducers, were also up-regulated following COLE therapy. Notably, COLE-treated cells exhibited higher expression levels of the NFKB1 gene compared to untreated cells [81,82]. Given NF-kappaB’s well-established role as a central activator of anti-apoptotic cascades in response to external stimuli or internal immunological responses, its increased expression following COLE treatment suggests a potential prosurvival mechanism underlying the observed effects [81,82].

## 7. Olive Oil Compounds and Acute Lymphoblastic Leukemia

A study conducted by Kitsati et al. [54] sheds light on the potential of olive oil compounds, particularly hydroxytyrosol, to modulate intracellular iron homeostasis and thereby influence redox-mediated signal transduction [54]. In their investigation, JURKAT cells, derived from a human T-lymphocytic cell line, were employed. Additionally, single-cell gel electrophoresis (comet assay) was utilized to categorize DNA damage into five classes [54].

Their findings suggest that the rapid increase in the acute phase protein ferritin observed upon exposure to H_2_O_2_ should be interpreted as a protective mechanism. This response indicates that cells promptly react to avert potential harmful consequences arising from the concurrent presence of elevated levels of labile iron and H_2_O_2_. Notably, pre-treatment with hydroxytyrosol (HTy), but not tyrosol (Ty), resulted in a reduction in H_2_O_2_-induced labile iron increase and ferritin expression. This underscores the essential role of the ortho-dihydroxy group for the beneficial action of HTy, possibly exerted at the levels of mRNA stability or translation [54]. Furthermore, HTy exhibited a remarkable selectivity in modifying the H_2_O_2_-induced MAPK phosphorylation pattern. While the rapidly induced ERK phosphorylation and early p38 phosphorylation remained unaffected, the late and sustained phases of JNK and p38 phosphorylation were reduced. This suggests that HTy has a unique effect that suppresses the rise in labile iron levels in the cytosol following H_2_O_2_ exposure. By removing iron from specific sites, HTy renders them insensitive to oxidation, thereby inactivating upstream kinases and preserving the activity of respective MAPK phosphatases under oxidative stress conditions. This intricate mechanism is crucial, as the actual level of MAPK phosphorylation is determined by the coordinated action of upstream MAP3K and MAP2K, along with the respective MAPK phosphatases. In contrast, the higher iron levels allow iron ions to bind to these specific sites in the absence of HTy (or in the presence of ineffective Ty), resulting in opposite effects [54]. The study suggests that any substance in the human diet capable of crossing biological membranes and chelating intracellular labile iron may potentially mitigate apoptotic redox signaling and H_2_O_2_-induced DNA damage. This inference is drawn from the research findings presented in this study [54].

According to Parra-Perez’s study, hydroxytyrosol use in T-cell acute lymphoblastic leukemia cell lines (JURKAT cells) results in cell cycle arrest. In particular, they observed a significant increase in G0/G1-phase cells compared to the control group and a significant decrease in the S phase [55]. The production of ROS and apoptotic mechanisms are both increased by hydroxytyrosol. They notice a large increase in cell death and a considerable decrease in cell differentiation in Jurkat cells by reducing the expression levels of KSR1 and cMyc, respectively [54]. The findings of the current investigation further demonstrated that HT markedly reduced the activity of the PI3K/AKT pathway in Jurkat cells. On the other hand, there was an increase in ERK1/2/MAPK levels. Since the Ras and PI3K pathways can interact through crosstalk between their downstream effectors, the up-regulation in the MAPK pathway was a result of the down-regulation in PI3K [55,83]. This action may be responsible for the greater levels of apoptosis, which were generated by HTy’s effects on caspase 9 levels and Bcl-2 levels [83]. 

## 8. Olive Oil Compounds and Multiple Myeloma

An interesting new research was investigated in recent years, based on the use of phenolic compounds, particularly hydroxytyrosol, in various human multiple myeloma (MM) cell lines. A study conducted by Todoerti et al. [56] demonstrates that HTOL (a synthetic fatty ester of natural hydroxytyrosol with oleic acid) reduces the viability of human myeloma cell lines (HMCLs) at low micromolar doses, even in the presence of bone marrow stromal cells (BMSCs), without exhibiting cytotoxicity towards healthy peripheral blood mononuclear cells (PBMCs) or B-lymphocytes [56,84,85,86,87]. Moreover, HTOL was found to down-regulate Interferon signaling pathways, which are pivotal in controlling various immune responses, both innate and adaptive [88]. Specifically, the study highlights the role of Interferon regulatory factor 4 (IRF4) as a lymphocyte transcription factor. IRF4’s N-terminal DNA binding domain (DBD) contains a helix–loop–helix motif crucial for identifying specific DNA sequences related to the interferon-stimulated response element [89]. Notably, IRF4 translocation to actively transcribed genomic areas in some multiple myeloma (MM) patients is associated with carcinogenicity and overexpression, yet it confers a survival benefit in MM without translocations or amplifications [56,89]. Several studies have underscored the essential role of IRF4 in MM cell survival, with genetic knockdown of IRF4 or targeting the tumor suppressor miR-125b being fatal to MM cells [90,91]. Downstream of IRF4, B-lymphocyte-induced maturation protein-1 (BLIMP-1) triggers apoptosis in MM cells when knocked down [92]. Additionally, IRF4 transactivates caspase-10, with loss of the caspase-10/cFlip heterodimer, resulting from IRF4 knockdown, leading to MM cell death [93]. Given these findings, IRF4 emerges as a promising therapeutic target for MM [94]. This study also highlights the significance of the IRF4-c-MYC axis in MM development, as c-MYC is overexpressed as the disease progresses and is associated with drug resistance to various therapies [94,95,96]. HTOL was found to block the IRF4 signaling pathway, leading to an arrest in the development of myeloma cells. Importantly, due to the higher expression of molecular targets identified by the IRF4-cMYC axis in MM cells, HTOL-based treatments exhibit low toxicity [56]. This study suggests that targeting the IRF4-c-MYC axis, which remains challenging due to the lack of clinically useful targeted treatments, may offer novel therapeutic approaches not only for MM but also for other c-MYC-dependent cancer types [56].

Another interesting study was performed by Juli et al. [57], who investigated the anti-tumor potential of oleacein, starting from the easily available natural oleuropein [57,97]. Following oleacein administration, they saw a significant rise in the acetylation of histones and acetyl-tubulin. This discovery demonstrates a novel histone deacetylase (HDAC) inhibitory activity of oleacein in MM. Oleacein, in fact, has the potential to suppress the transcriptional activity of HDACs by targeting Sp1, a recognized transactivator of the HDACs promoter. The caspase 8 inhibitor Z-IETD-FMK was able to reverse the effects of oleacein on Sp1 down-regulation [57]. Additionally, Sp1, a pleiotropic transcription factor with carcinogenic potential in human malignancies [98], was found to have a detrimental impact on miRNA expression [99]. It is crucial to note that while some miRNAs, like miR-21, miR-125a-5p, miR-17-92, and the miR-221 cluster are highly expressed in multiple myeloma and function as oncogenes, others, like miR-22, miR-29b, and miR-125b, act as tumor suppressors [91,100,101,102,103,104]. Oleacein caused the overexpression of two tumor suppressive miRNAs, miR-22 and miR-29b, which are known to be negatively regulated by this transcription factor, which is consistent with Sp1 inhibition [103,104,105,106]. These results highlight oleacein’s capacity to activate a tumor-suppressive miRNA network, which probably contributes to its cytotoxicity against MM cells. Additionally, this research has demonstrated that oleacein may boost the anti-tumor effects of carfilzomib or bortezomib. Notably, oleacein synergistically increased the in vitro cytotoxicity of carfilzomib, leading to superior Sp1 and HDAC down-regulation and a rise in MM cell death [57].

## 9. Olive Oil Compounds and CLL: A Pilot Randomized Trial

Rojas Gil et al.’s study [58] focused on the potential use of olive oil in diet plans for CLL patients in Rai stages O to II who did not meet the requirements to begin chemotherapy. In this study, they tried to document cell apoptosis using both specific hematological markers, such as white blood cells and hemoglobin, and specific molecular markers of apoptosis (ccK18 Survivin and Apo1-Fas). The patient enrollment process was divided into two stages. In the first, the patients consumed two types of olive oil: High OC/OL-EVOO and Low OC/OL-EVOO, which are both high in oleocanthal and oleacein. In the second, they solely ingested High OC/OL-EVOO [58]. According to the current study, High OC/OL-EVOO may be able to cause apoptosis and cell cycle arrest in those with early-stage CLL. The serum protein expression of the anti-apoptotic protein Survivin decreased and that of the apoptotic markers ccK18 and Apo1-Fas increased throughout the dietary intervention (both DI1 and DI2), according to research [58]. In the present study, PMBC protein expression analysis revealed a decrease in the protein expression of Cyclin D and Survivin and an increase in the protein expression of p21WAF1, indicating a detrimental effect on the cell cycle with Alto OC/OL-EVOO. Additionally, a rise in the isolated PMBC’s apoptotic rates during DI2 was detected by the TUNEL assay. Additionally, there was a positive association between the WBC at the conclusion of the food intervention and the final level of p21 protein expression [107,108,109]. It was demonstrated for the first time that oral administration of a daily dose of 25 mg oleocanthal and oleacein through consumption of 40 mL of EVOO could be beneficial for CLL patients, inducing the death of their cancer cells and enhancing their metabolism [58], though this pilot trial was constrained by the number of participants and the duration of the intervention.

## 10. Conclusions and Future Perspectives

Cancer is one of the leading causes of mortality worldwide, and research reveals that in 2030, the number of new cancer cases will be about 26 million with an annual 17 million cancer deaths [110,111]. The expected increase will be largest in low- and medium-income countries due to different risk factors such as sedentary lifestyle, tobacco smoke, aging of populations, and dietary habits [110]. Despite considerable efforts in prevention and therapy, the incidence and mortality rates for most forms of cancer remain high [110,112].

Nowadays, the field of research is largely directed towards new studies on the use of natural compounds and the science of nutraceuticals. Different studies have documented that different natural compounds can have important contributions in the treatment of both solid tumors and hematological neoplasms. In recent years, studies on the possible use of officinalis plants in hematology’s clinical practice have increasingly expanded to be able to minimize the side effects and current therapies used. Some compounds studied in different clinical studies, including St. John’s Wort and *Rosmarinus officinalis* L., have demonstrated a potential role in inhibiting cell proliferation through the control of gene expression and caused programmed cell death and associated ROS/nitrogen (RNS) generation [113,114].

**Figure 3:**
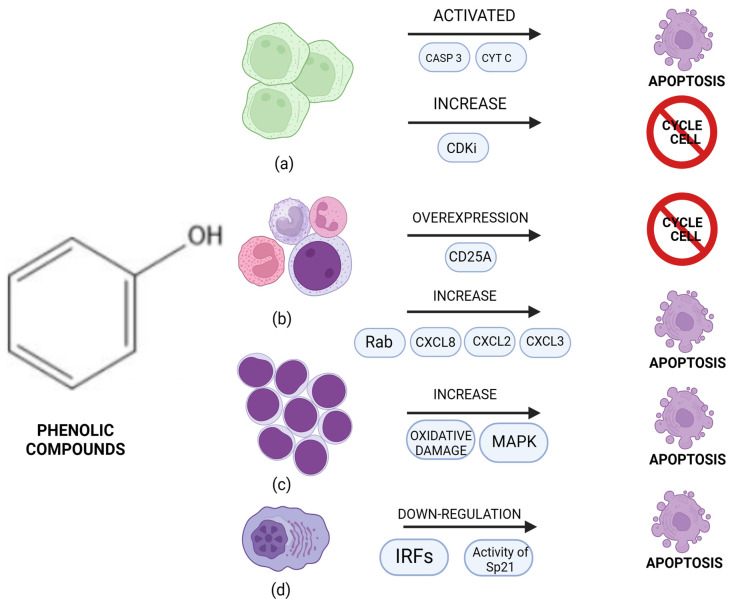
Effect of olive oil compounds on hematological malignancies: (**a**) promyelocytic leukemia; (**b**) chronic myeloid leukemia; (**c**) acute lymphoblastic leukemia; (**d**) multiple myeloma. “Created with BioRender.com (accessed on 1 February 2024)”.

Our investigative pursuits have been intricately woven around the exploration of compounds sourced from olive oil and phenolic constituents [53,54,56,59] (Figure 3). These phenolic entities, as elucidated by the studies analyzed by us, exhibit a remarkable panoply of effects, underscoring their potential as potent agents in combatting neoplastic cell proliferation. The multifaceted actions of these phenolic compounds are indeed noteworthy. They demonstrate a nuanced capacity to intervene at various checkpoints of the cell cycle, effectively thwarting aberrant cell division processes. Furthermore, through their intricate modulation of cytokine activity, they orchestrate a finely tuned homeostatic balance that meticulously regulates the unbridled proliferation of malignant cells. Moreover, their induction of reactive oxygen species serves as a formidable trigger for pro-apoptotic mechanisms, thus catalyzing the demise of cancer cells.

However, it behooves us to acknowledge the inherent limitations within our research milieu. The relatively modest number of studies scrutinized, coupled with constraints such as sample size and intervention duration, underscores the need for judicious interpretation of our findings. To address this, further collaborative efforts are warranted to increase the number of studies and enhance the sample size, ensuring a more comprehensive representation of the population under study. Moreover, extending the duration of interventions could provide valuable insights into the long-term effects of natural compounds and nutraceuticals on neoplasms.

Furthermore, exploring synergistic interactions between these compounds and conventional chemotherapy regimens, particularly within the purview of hematological neoplasms, presents an opportunity for future inquiry. Conducting combined studies that evaluate the efficacy of integrated treatments may shed light on novel therapeutic approaches.

In addition, rigorous clinical trials are imperative to evaluate the efficacy and safety of natural compounds in treating neoplasms. These trials should be meticulously designed and executed to provide robust evidence of treatment effectiveness.

Standardizing extraction methodologies is another crucial step forward. By establishing consistent extraction protocols, we can ensure the reproducibility and reliability of results across studies, minimizing variations attributed to extraction methods.

Lastly, promoting translational research is essential. Bridging the gap between preclinical discoveries and clinical applications requires collaboration among researchers, clinicians, and industry stakeholders to translate scientific findings into tangible therapies and treatments for patients.

In conclusion, while our findings highlight the potential of natural compounds in combating neoplastic cell proliferation, addressing these limitations and implementing the aforementioned solutions will be pivotal in advancing our understanding and treatment of neoplastic pathologies. Integrating concepts such as increasing collaborative research efforts, promoting data sharing, investing in innovative technologies, enhancing awareness and education, and supporting interdisciplinary research can further strengthen our approach towards combating cancer comprehensively and effectively.

## Figures and Tables

**Table 1:** Synthesis of the mayor studies shown following.

Purpose of the Study	Experimental Model and Phenolic Compounds	Mechanism of Action	Ref.
Hydroxytyrosol, a natural molecule occurring in olive oil, induces cytochrome c-dependent apoptosis	Human promyelocytic leukemia cells (HL60) and 3,4-DHPEA([3,4-dihydroxyphenylethanol,Hydroxytyrosol)	The presence of two ortho-hydroxyl groups causes the activation of cytochrome C and CASPASE 3 to determine apoptosis	[52]
Virgin olive oil phenols inhibit proliferation of human promyelocytic leukemia cells (HL60) by inducing apoptosis and differentiation	Human promyelocytic leukemia cells (HL60) and 3,4-DHPEA([3,4-dihydroxyphenyl-ethanol,Hydroxytyrosol)	The compounds cause cell cycle arrest in GO/G1 phase, and otherwise increase CDk inhibitors and production of H202 in culture	[48]
Olive (*Oleaeuropaea*) leaf extract induces apoptosis and monocyte/macrophage differentiation in human chronic myelogenous leukemia K562 cells: insight into the underlying mechanism	Human chronic myelogenous leukemia (K562 cells) and COLE (Chemlali olive leaf extract)	COLE causes the first-time arrest in the G0/G1 phase, followed by G2/M. This process is mediated by the overexpression of CD25A gene. Otherwise, it showed an increase in CD14 on cell surfaces and of genes correlated with apoptosis such asAP1G1 and Rab proteins, CXCL8 (IL8), CXCL2, and CXCL3	[53]
Hydroxytyrosol inhibits hydrogen peroxide-induced apoptotic signaling via labile iron chelation.	Human T-lymphocytic cell line (JURKAT cell) and 3,4-DHPEA([3,4-dihydroxyphenyl-ethanol,Hydroxytyrosol)	The two ortho-hydroxyl groups of HTy cause the production of H202 followed by an increase in labile iron and ferritin that induce MAPk pattern phosphorylation.	[54]
Involvement of the PI3K/AKT Intracellular Signaling Pathway in the AntiCancer Activity of Hydroxytyrosol, a Polyphenol from *Olea europaea*, in Hematological Cells and Implication of HSP60 Levels in Its Anti-Inflammatory Activity	Human T-lymphocytic cell line (JURKAT cell) and 3,4-DHPEA([3,4-dihydroxyphenyl-ethanol,Hydroxytyrosol)	The phenolic compound causes arrest in the G0/G1 phase of the cell cycle. Otherwise, HTy reduced the activity of the PI3K/AKT pathway and increased ERK1/2/MAPK	[55]
OleilHydroxytyrosol (HTOL) Exerts Anti-Myeloma Activity by Antagonizing Key Survival Pathways in Malignant Plasma Cells.	Human multiple myeloma cell lines and HTOL (a synthetic fatty ester of natural hydroxytyrosol with oleic)	HTOL causes a down-regulation of Interferon signaling. IRFs, particularly IRF4, are over-expressed in MM cells.	[56]
Anti-tumor Activity and Epigenetic Impact of the Polyphenol Oleacein in Multiple Myeloma	Human multiple myeloma cell lines and Oleacein	Oleacein causes an increase in apoptosis through epigenetic mechanisms. It determines the suppression of activity of Sp21, a transcriptor recognized as a HDAC promoter	[57]
The Effect of Dietary Intervention With High-Oleocanthal and Oleacein Olive Oil in Patients With Early-Stage Chronic Lymphocytic Leukemia: A Pilot Randomized Trial	In vivo patient with CLL and Oleocanthal and Oleacein	Oleocanthal and Oleacein determine an increase in some proapoptotic markers such as ccK18 and Apo1-Fas	[58]

## Data Availability

No new data were created or analyzed in this study. Data sharing is not applicable to this article.
